# Evolution of an Agriculture-Associated Disease Causing *Campylobacter coli* Clade: Evidence from National Surveillance Data in Scotland

**DOI:** 10.1371/journal.pone.0015708

**Published:** 2010-12-15

**Authors:** Samuel K. Sheppard, John F. Dallas, Daniel J. Wilson, Norval J. C. Strachan, Noel D. McCarthy, Keith A. Jolley, Frances M. Colles, Ovidiu Rotariu, Iain D. Ogden, Ken J. Forbes, Martin C. J. Maiden

**Affiliations:** 1 Department of Zoology, University of Oxford, Oxford, United Kingdom; 2 Department of Medical Microbiology, University of Aberdeen, Aberdeen, United Kingdom; 3 School of Biological Sciences, University of Aberdeen, Aberdeen, United Kingdom; 4 Nuffield Department of Clinical Medicine, University of Oxford, Oxford, United Kingdom; Royal Tropical Institute, Netherlands

## Abstract

The common zoonotic pathogen *Campylobacter coli* is an important cause of bacterial gastroenteritis worldwide but its evolution is incompletely understood. Using multilocus sequence type (MLST) data of 7 housekeeping genes from a national survey of *Campylobacter* in Scotland (2005/6), and a combined population genetic-phylogenetics approach, we investigated the evolutionary history of *C. coli*. Genealogical reconstruction of isolates from clinical infection, farm animals and the environment, revealed a three-clade genetic structure. The majority of farm animal, and all disease causing genotypes belonged to a single clade (clade 1) which had comparatively low synonymous sequence diversity, little deep branching genetic structure, and a higher number of shared alleles providing evidence of recent clonal decent. Calibration of the rate of molecular evolution, based on within-species genetic variation, estimated a more rapid rate of evolution than in traditional estimates. This placed the divergence of the clades at less than 2500 years ago, consistent with the introduction of an agricultural niche having had an effect upon the evolution of the *C. coli* clades. Attribution of clinical isolate genotypes to source, using an asymmetric island model, confirmed that strains from chicken and ruminants, and not pigs or turkeys, are the principal source of human *C. coli* infection. Taken together these analyses are consistent with an evolutionary scenario describing the emergence of agriculture-associated *C. coli* lineage that is an important human pathogen.

## Introduction

Members of the genus *Campylobacter* are among the most common cause of bacterial gastroenteritis worldwide. The species that are principally responsible for human disease, *Campylobacter jejuni* and *Campylobacter coli* [1], cause sporadic infection [2] and occasional outbreaks [3] usually associated with consumption of contaminated meat, poultry, water and contact with animals [4], [5]. Although *C. coli* is responsible for fewer food-borne illnesses than *C. jejuni*, the impact of *C. coli* is still substantial: of approximately 340000 annual cases of campylobacteriosis in the UK and 2.5 million in the US [6], [7], [8], approximately 10% are caused by *C. coli* [9]. Therefore, based upon recent estimates, *C. coli* infection has an annual cost of £50 million in the UK [10] and $800 million in the USA [11] but despite the economic importance of this pathogen, most *Campylobacter* research focuses upon *C. jejuni*.

Multi-locus sequence typing (MLST) is a molecular typing technique that has enhanced studies of the population structure and epidemiology of *Campylobacter* [12]. An MLST scheme has been developed for *C. coli* [13], [14] which characterizes allelic orthologues of the same seven housekeeping gene loci as the original *C. jejuni* MLST system [12]. The inherent reproducibility of this nucleotide sequence based method and the ability to compare data from different laboratories has enabled the assembly of large archives of isolate genotype data [15] which provide a valuable resource for analysing the epidemiology and evolution of these organisms.

To date, studies of the genetic structure of *C. coli* populations have suggested that there is less genetic diversity than in *C. jejuni* isolate collections from a comparable sample frame [13], [14], [16]. However, sufficient variation in MLST alleles exists to identify correlation with the animal host from which the isolate was sampled [17], [18] and recent analyses have exploited this host-genotype relationship to investigate potential disease reservoirs. By describing spatiotemporal patterns and lineage associations in animal hosts [19], [20] and using computer models for the attribution of clinical isolates to source [21], [22], [23], [24] these studies consistently link genotypes from clinical isolates with the greatest probability to ruminant and especially chicken (55–80% of isolates) sources. In contrast to *C. jejuni*, where there is little evidence of deep branching phylogenetic structure, *C. coli* is divided into three clades [25]. There is evidence that the clades are associated with sample source, with clades 2 and 3 more common in environmental waters [26], but little is known about the clades, for example when they arose, what is their ecological significance and, in particular, how they relate to clinical infection caused by *C. coli*.

Using isolate genotype data from a national survey, the *Campylobacter* MLST project in Scotland - CaMPS (2005/6), we aimed to investigate the disease causing *C. coli* by analysing the genetic structure of isolates from a variety of sources including clinical infection, farm animals and the environment and using population genetic techniques to characterise the lineages associated with human disease. *C. coli* was studied by investigating (i) the phylogenetic relationships among isolates (ii) the intrinsic genetic differences between *C. coli* and *C. jejuni* lineages, (iii) the quantitative attribution of clinical isolates to different sources, and (iv) the rate of molecular evolution and the time of species and clade divergence. Taken together, these analyses provide evidence for an evolutionary scenario that describes the emergence of clinically important *C. coli*.

## Results

### Clinical and potential source isolates

There were 5,674 isolates from confirmed clinical cases of campylobacteriosis, received from 15 health board regions in Scotland between July 2005 and September 2006. Of these, 427 isolates were excluded because they comprised mixed cultures, incomplete typing or other *Campylobacter* species such as *C. lari* and *C. upsaliensis*. Of the remaining clinical isolates (5247), MLST confirmed that 4747 were *C. jejuni* and 500 were *C. coli*. In addition to isolates from human disease, a total of 200 *C. coli* isolates from samples from potential source populations were typed at 7 loci. These were augmented with archive data (1023 isolates) from published sources [13], [17], [22], [26], [27], [28], [29] to give a total of 1223 isolates from potential sources. Isolates were grouped by source/host animal to give isolate datasets for phylogenetic and attribution analysis from 98 cattle, 54 sheep, 514 chicken, 380 swine, 110 turkey and 67 riparian (water fowl and environmental waters) sources (Table S1).

### Genetic diversity

There were a total of 451 STs, 103 from clinical isolates and 393 from other sources. The 10 most common STs in clinical (ST-827, ST-825, ST-1774, ST-855, ST-829, ST-1614, ST-872, ST-962, ST-828, ST-1773) and non-clinical (ST-827, ST-825, ST-1068, ST-829, ST-855, ST-854, ST-1101, ST-1614, ST-1017, ST-962) datasets accounted for 72% and 37% of genotypes respectively. Two clonal complexes were present, defined as in the standard definition as groups of STs that share 4 or more alleles in common with the central genotype. Eighty-one percent and 1% of clinical isolates belonged to the ST-828 complex and the ST-1150 complexes respectively and 52% and 4% of non-clinical isolates belonged to these complexes. The remainder of isolates did not belong to a known clonal complex. The clonal complex structure within *C. jejuni* is greater compared to data sets of a similar magnitude from comparable sources [13], and this allows the identification of clonal complexes with different levels of host association [20], [30]. Similar association analysis was not possible for *C. coli* at the clonal complex level but STs belonging to the ST-828 complex have previously been recovered from clinical disease isolates and from agricultural sources [13], [17]. There was some variation in allelic diversity by locus (Table S2) but it was generally low, with the total number of STs (451) approximately equal to the number of alleles (410) suggesting that the variation in genotypes results more from re-assortment of existing alleles than generation of new ones by point mutation which would give more alleles per locus. The clinical isolate population had different genetic properties (lower diversity) with the mean number of alleles per locus (13) lower than in the non-clinical data (55).

### Clonal frame genealogy

**Figure 1:**
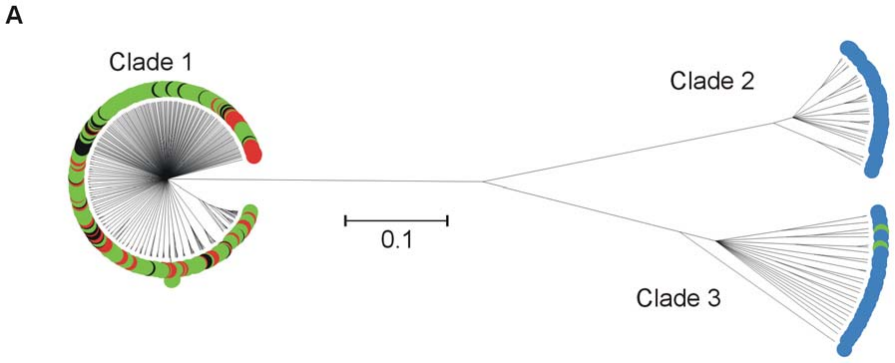
ClonalFrame genealogy of 7-locus MLST data from *C. coli* genotypes from clinical, animal faeces, food and environmental samples. STs from farm only (green), riparian only (blue), clinical only (red) and farm and clinical (black) sources are indicated by different colours.

The genealogy determined using CLONALFRAME showed a high degree of genetic structuring in isolates sampled from clinical infection animal sources and the riparian environment (Figure 1). The 3-clade structure that has previously been described [25] was evident. Comparison of genotypes from clinical infection with this genealogy demonstrated that all of the cases of human *C. coli* infection were caused by lineages belonging to clade 1 (Table 1). Eighty-four percent of STs from clade 1 belonged to the ST-828 clonal complex.

**Table 1:** Genotypic diversity of *C. coli* clades from different sources.

Clade	Number of STs	Number of STs from each source		
		Clinical	Farm animal	Riparian
1	393	103	335	0
2	31	0	0	31
3	27	0	2	25

### Phylogenetic congruence

**Figure 2:**
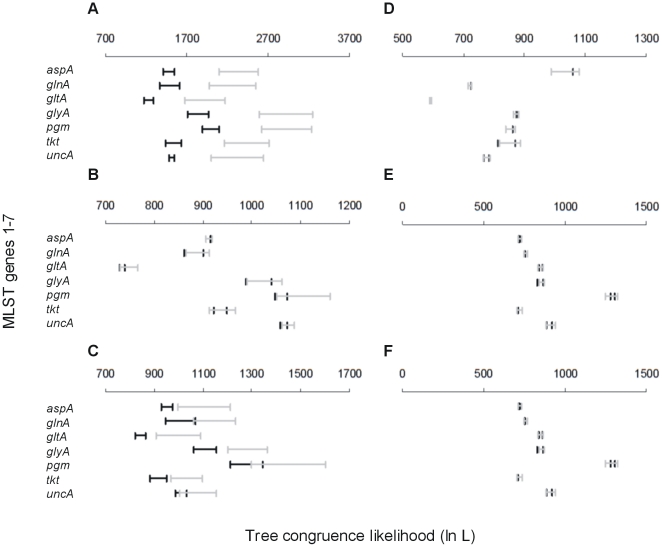
Maximum likelihood (ML) analysis of congruence in *Campylobacter jejuni* and *Campylobacter coli*. The ML tree of each MLST locus is compared with the ML trees from the other six loci for: (A) *C. coli* clades 1–3, (B) *C. jejuni*, (C) *C. coli* and *C. jejuni* (D) *C. coli* clade1, (E) *C. coli* clade 2 and (F) *C. coli* clade 3. The tree congruence likelihood (ln L) range is shown with horizontal bars for each locus (black) and for 200 trees of random topology (grey) for *aspA*, *glnA*, *gltA*, *glyA*, *pgm*, *tkt* and *unc* respectively from top to bottom on each graph. If the ln L range falls outside the range calculated for random trees there is more congruence among the ML trees than expected by chance.

ML trees were determined for each of the 7 MLST loci for *C. jejuni* and *C. coli* (clades 1–3), and *C. jejuni* and *C. coli* combined (data not shown). The topology of the trees for *C. jejuni* sequences showed no evidence of congruence, with alleles frequently changing position amongst trees. For the combined *C. jejuni/C. coli* trees there was congruence, partitioning alleles in accordance with species. Similarly, with *C. coli* there was congruence within the three clades. Visual representation of congruence indicated that within *C. coli* STs, alleles are more likely to be associated with those from the same clade. Quantitative analysis of congruence was performed using the SH test on the ML trees for combined *C. coli* data, *C. jejuni/C. coli*, *C. jejuni*, and the three *C. coli* clades separately (Figure 2). Within *C. jejuni* there was no evidence of congruence with the likelihood values (-ln L) for all of the single locus trees within the range of -ln L values generated for random trees (Figure 2B). This suggests extensive recombination. The -ln L for combined *C. jejuni/C. coli* trees provided evidence of tree congruence for *aspA, gltA, glyA* and *tkt* (Figure 2C). This is expected as the likelihood of one single locus tree predicting another is high when they share a distinct two-species distribution. Within *C. coli* the congruence between single locus trees, suggested by ST restriction within clades, was confirmed with likelihood values for congruence between competing ML trees outside of the –ln L values for random trees for all 7 MLST loci. This suggests relatively low levels of recombination between clades. Analysis within individual *C. coli* clades showed no evidence of congruence indicative of recombination within clades.

### Molecular clock estimates of clade divergence

**Figure 3:**
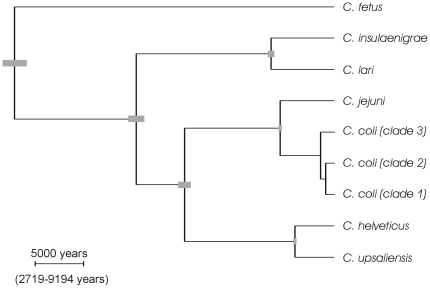
Phylogeny of estimated divergence times in the genus *Campylobacter* using BEAST. Error bars, in grey, associated with each node indicate relative uncertainty in node height. Calibration of the scale bar was based on intraspecific variation in *Campylobacter jejuni* and uncertainty associated with the molecular clock calibration is represented by a 95% CI below the scale bar. Empirical and indirect [35] estimates of the rate of molecular evolution would calibrate the same scale bar at 42,200 (95% CI 2,690–661,000) and 7,600,000 years respectively.

The *C. jejuni* population from the 3 year longitudinal study [21] that was used to calibrate the tree contained sufficient levels of mutation, recombination or coalescence events to estimate the timescale of the genealogy (*Neg*), where *Ne* is the effective population size and *g* is the generation length. The rate of molecular change was analysed using the importance sampler [31] and the results of the three alternative datasets were merged to produce a model average over the datasets. There was negligible uncertainty in the tree topology (Figure 3), and the topology was as expected from separate analyses [32]. The uncertainty in the scale bar, which represents uncertainty in the calibration of the molecular clock, was (2719–9194 years) for a scale bar of length 5000 years. The point estimates for the divergence of the different *Campylobacter* lineages were consistent with previous estimates [32] and placed the divergence of *C. coli* and *C. jejuni* at 6429 (95% CI, 6280–6579) years ago with *C. coli* clade 3 diverging approximately 1684 (95% CI, 1659–1709) years ago and clades 1 and 2 diverging approximately 1023 (95% CI, 1005–1041) years ago (Table 2).

**Table 2:** Estimates of the time of Phylogenetic splits in the genus *Campylobacter*.

	95% CI		
Split	Point estimate (years)	Relative uncertainty (years)	Total uncertainty* (years)
*fetus – jejuni*	38269	36824–39770	20198–73764
*lari – jejuni*	23713	22830–24630	12499–45597
*helveticus – jejuni*	17907	17155–18691	9403–34645
*insulaenigrae – lari*	7614	7252–7994	3977–14825
*coli – jejuni*	6479	6380–6579	3497–12193
*helveticus – upsaliensis*	4729	4575–4889	2509–9069
*coli* (clade3) *– coli* (clades 1 &2)	1684	1659–1709	903–3145
*coli* (clade 1) *– coli* (clade 2)	1023	1005–1041	549–1924

*Total uncertainty in each of the split times including uncertainty in the calibration of the molecular clock based on the intraspecific genetic variation in *C. jejuni*.

The traditional method for dating recent bacterial evolution [33], [34] is to calibrate the rate of sequence divergence relative to the split of *E. coli* and *Salmonella typhimurium* which Ochman and Wilson estimated at 120–160 million years ago (Ma), based on 1% divergence in the 16S rRNA gene per 50 million years [35]. By this estimate the *C. jejuni-C. coli* split would have occurred approximately 10 Ma [32], very different to the estimate (6479 years) using our method, based on intra-specific variation. By our estimate, speciation is occurring over thousands rather than millions of years. The root of the tree was dated at 38,269 years ago (95% CI 20198–73764) and the speciation rate (λ) was 0.054 (95% CI 0.019–0.14) per lineage per 1000 years [36]. Estimates of mutation rate, µ = 0.029, (95% CI 0.016–0.053), the transition-transversion ratio, κ = 2.86 (95% CI 2.47–3.31), and *d*_N_/*d*_S_ ratio, ω = 0.011 (95% CI 0.009–0.014) were in good agreement with previous estimates for this genus [32]. To enable comparison of our molecular clock calibration based on intraspecific variation with other estimates, the time represented by the scale bar in figure 3 was determined using other calibration methods. With an empirical estimate, based on published generation times [37], [38] and genomic mutation rates [39] for *C. jejuni*, the scale bar would represent a period of 42,200 years (95% CI 2,690–661,000) and with the Ochman and Wilson method [35] the scale bar would represent 7.6 My [32].

### Attribution to putative source

Isolates from known sources were used to test the limitations of the attribution model as previously described [22]. Random subsets of the comparison datasets for all putative source populations were used for self-assignment. Test sets of 50% of the swine, ruminant, chicken, turkey and riparian isolates were assigned to host source based on a reduced training set, and the AI model assigned them with 94%, 89%, 95%, 92%, 88% probability to the correct source: swine, ruminant, chicken, turkey and riparian sources respectively. For each clinical isolate the probability of assignment to each potential source was calculated and the sum of these probabilities was used to determine the percentage of all clinical isolates that are attributed to each source. The clinical *C. coli* isolates were attributed to source in the following percentages: 57% to poultry, 41% to ruminant, 1% to swine, 0.5% to turkey and 0.5% to riparian sources.

## Discussion

The genealogical reconstruction of genotypes from diverse sources demonstrated the clade structure and revealed that all of human *C. coli* disease cases were caused by STs belonging to clade 1. The 3-clade structure, and its relationship to disease, is poorly understood but for it to arise and be maintained requires differentiated gene pools. The simplest explanation for this is a general reduction in the overall level of recombination but there is evidence of frequent recombination within each clade (Figure 2), and therefore it is likely that recombinational barriers are involved in clade divergence. Three broad classes of barrier can be described, (i) mechanistic barriers - imposed by the homology dependence of recombination [40] or other factors promoting DNA specificity such as restriction/modification systems [41]; (ii) ecological barriers - a consequence of physical separation of bacterial populations in distinct niches; (iii) adaptive barriers - implying selection against hybrid genotypes [42]. The relative importance of these three different classes of barrier is unclear but evidence from interspecies recombination between *C. coli* and *C. jejuni* [25] is consistent with the involvement of factors such as physical proximity, a shared vector (bacteriophage) infecting both lineages, or niche adaptation playing a role.

*C. coli*, clade 1 has several genetic characteristics that distinguish it from other lineages within *C. coli*. First, this clade has relatively low synonymous sequence diversity in MLST genes (mean D_s_ of 0.006 per nucleotide) compared to *C. jejuni* (0.016) and clades 2 (0.008) and 3 (0.013). The sequence diversity is higher than that of organisms commonly described as genetically monomorphic (Ds of <0.0002), for example enteric *Salmonella Typhi* or *Yersinia pestis* [43], but is still comparatively low indicating a relatively recent genetic bottleneck. Second, on a tree based on concatenating multiple genetic regions from each strain, in this case MLST data, there is little evidence of deep genetic structure that would indicate long periods of independent evolution of clade 1 (or clade 2). Third, there is more evidence of genetic exchange within *C. coli* clade 1 as STs often share the same allele at individual MLST loci and as in other bacterial species, this sharing of the majority of alleles is strong evidence of recent clonal descent.

The importance of recombination in generating variation also varies among the clades. The relative importance of the re-assortment of existing alleles (recombination) and the generation of new ones (mutation), in producing variation can be provided by comparison of the number of alleles at each locus, ranging in this study from 120 for *aspA* to 221 for *pgm*, with the number expected for the number of STs (1441) if point mutation generating novel alleles were responsible for the diversity (1441/7 = 206). If the observed number of alleles is lower than this expected value then recombination is involved in generating diversity. Using this method the ratio of observed to expected alleles was 0.5 for *C. coli* clade 1 and 0.7 for *C. jejuni* compared to 2.6 and 4.9 for clades 2 and 3 respectively. This indicates that recombination is 5–10 times more important in generating diversity in *C. coli* clade 1 than in the other clades. Recent work that uses combined population-genetic microevolutionary models demonstrates that recombination generates twice as much diversity as de novo mutation in *Campylobacter* and has a fundamental role in the evolution of this genus [32]. Our findings for *C. coli* clade 1 are consistent with these results but demonstrate that the importance of recombination can vary within subgroups of the same species (clades 2 and 3).

The numerical dominance of clade 1 *C. coli* genotypes from clinical disease can be explained, without consideration of intrinsic differences in the ability to infect humans, as a reflection of the dominance of isolates from this clade in disease reservoirs and food chain sources. Like *C. jejuni*, there are limitations on how well *C. coli* lineages can be attributed to source because, while there are host-associated alleles [17], [30], there is also considerable niche overlap with the same alleles and STs found in isolates from different farm hosts [20], [22]. However, alleles from STs clustered into clade 1 are distinct from those in clades 2 and 3 and this level of genetic differentiation allows source attribution. This shows that poultry and ruminants and not pigs and turkeys (clade 1) or the riparian environment (the source of most clade 2 and 3 isolates), are the most likely source of human *C. coli* infection. This is consistent with evidence of the source of human *C. jejuni* infection [21], [22], [44]. In evolutionary terms, agriculture is a new niche and the co-infection of animals including chicken and ruminants by similar strains, despite the differences in the biology of their digestive tracts, suggests that this niche has acquired specifically adapted lineages of bacteria, rather than sharing a common gene pool with a pre-existing natural reservoir. Agricultural environments are highly unusual in terms of host diet, genetic and age structure, density and many other details of habitation. It appears that this novel niche has been recently colonised by clade 1 *C. coli* as indicated not only by evidence of a smaller historical population size but also by the nature of recently recombined *C. jejuni* alleles found in *C. coli* clade 1. These alleles are typical of those found in *C. jejuni* from farm sources and are almost all identical to those found in the donor species, indicating that the imports occurred recently enough to have not accumulated mutations [25].

Determining the timescale of the evolutionary divergence of the three *C. coli* clades provides a context for understanding the link between ecological factors, such as the domestication of the host niche, and the population genetic structure. However, there is some debate about the rate of bacterial evolution because unlike multicellular eukaryotes they do not leave a morphologically informative fossil record and, unlike viruses, they do not mutate fast enough for evolution of natural populations to be easily measured in real time. Indirect methods calibrate the molecular clock in bacteria by cross-referencing events that can be dated. For example, the common ancestor of mitochondria and their closest living bacterial relatives [35] or cospeciation (of bacterial endosymbionts) with invertebrate hosts for which a fossil record is available [45]. Estimates based on such methods have been widely used [33], [34], [46], [47] but they can conflict with empirical approaches that are based on laboratory measurements of generation lengths and mutation rates [48], [49].

Dating *Campylobacter* evolution with reference to indirect methods for estimating the split of *E. coli* and *S. typhimurium* [35], would place the divergence of the three *C. coli* clades at approximately 2.5 million years ago. This is incompatible with the hypothesis that agriculture played a part in the divergence of the lineages because modern farming did not begin until around ten thousand years ago (in the Middle East), and was not common throughout Europe until much later (5,000–3,000 BC) [50], [51], [52]. However, the more recent estimate for the divergence of the clades, based on intra-specific variation in longitudinally sampled *C. jejuni* isolates, places the divergence of the *C. coli* clades at less than 2,500 years ago. This is consistent with the introduction of agriculture having had an effect upon the evolution of the genus *Camplylobacter*, for example by introducing novel opportunities for pathogens to expand into new host species and to spread rapidly to new territories. There are several potential causes for the conflict between short and long term estimates of bacterial evolution and the debate continues but an increasing number of studies support the theory of a far more rapid rate of bacterial evolution [32], [53], [54], [55]. Furthermore, the impact of agriculture upon bacterial evolution has been demonstrated in *Staphylococcus aureus* where the majority of isolates from chickens are the descendants of a single human-to-poultry host jump that occurred approximately 38 years ago (range, 30 to 63 years ago) in Poland [56]. This demonstrates the strong global effect that agriculture can have on bacterial evolution and may inform understanding of the population genetic structure of *Campylobacter* in the context of relatively rapid evolution.

Taken together the population genetic structure, source attribution of isolates and molecular clock calibration for clade divergence are consistent with an evolutionary scenario where a lineage within the *C. coli* has diverged into an agricultural niche. This may be related to intrinsic genetic differences associated with adaptation and the observation that some *C. jejuni* strains are more similar to particular strains of *C. coli* in gene content than they are to other strains of *C. jejuni* [57] supports this, but genome-wide studies of patterns of exchange are necessary to investigate the nature of barriers to gene flow and the consequences of recombination for ecological adaptation. Subtypes belonging to the agricultural *C. coli* lineage (clade 1) are responsible for the majority of human disease. It remains possible that there are genomic differences associated with pathogenicity but these are not required to explain the over representation of this clade among human disease cases. The most likely reason for the dominance of this clade in human disease is that it is an agricultural lineage and human disease is directly linked to food animals in the food chain.

## Materials and Methods

### Ethics statement

Ethical approval (reference: 05/S0802/151) for the collection of the samples and information used in this project was obtained from Grampian Local Research Ethics Committee (Summerfield house, Aberdeen, UK). This was in accordance with government agreements for research ethics committees (July 2001) and in compliance with the standard operating procedures in the UK. Specimens were collected from all 28 NHS clinical diagnostic laboratories in Scotland that agreed to participate. Archived isolate information included submitting laboratory, specimen number and date of collection. In accordance with Grampian Local Research Ethics Committee approval, patients were informed of the survey and had the option to decline. As no information that would allow identification of the patient was collected, individual patient consent was not required from patients that did not decline.

### Microbiology

The structured sampling and isolation procedure was carried out as described previously for the *Campylobacter* MLST project in Scotland (CaMPS)[19], [20], [22], [24]. In brief, clinical stool isolates (5674), animal faeces samples (2644) and food samples (394) were collected to represent all reported clinical cases of campylobacteriosis in the 15 health boards in Scotland (July 2005–September 2006) as well as a wide range of potential sources including contaminated food, animal faeces and the environment over several geographical areas and time periods. Twenty-five grams of food and fresh faecal (mammalian) and <5 g avian faecal samples were homogenised (1∶9) in *Campylobacter* enrichment broth [58] and plated (0.1 ml) onto charcoal cefoperazone deoxycholate (CCD, CM0739, Oxoid, UK) agar. The presence of *Campylobacter* was determined by incubating the remaining enrichment broth microaerobically (2% H_2_, 5% O_2_, 5% CO_2_, balance N_2_) 100-ml volumes of nutrient broth base (Mast, Bootle, UK) with 5% horse blood, growth supplement (Mast Selectavial SV61), amphotericin (2 µg/ml), cefoperazone (15 µg/ml) and trimethoprim (10 µg/ml) at 37°C. After 6–8 h enrichment, two additional antimicrobials (polymixin B, 2500 IU/litre, and rifampicin, 5 µg/ml) were added, and the broths were incubated for a further 5 days. Enrichment broths (0.1 ml) were plated, after 2 and 5 days, on CCD agar (Oxoid, UK) and incubated microaerobically at 37°C [59]. Gram Stain, Microscreen latex agglutination (Microgen, Camberley, UK) and PCR amplification - with primers specific for either *C. jejuni* or *C. coli* pgm genes [12]- were used to presumptively identify *Campylobacter*. Individual isolates were stored at −80°C in nutrient broth with 15% (v/v) glycerol.

### Multilocus sequence typing (MLST)

DNA was extracted from isolates recultured microaerobically at 37°C (for 48 h) with a CHELEX resin method (BIO-RAD, USA) as previously described [22]. A high throughput 7-locus MLST protocol was used, based upon a 2-phase robotic system for PCR of template DNA arrays and amplification products using published primers, reagent concentrations, template purification protocols and cycle parameters [12], [14], [58]. This process is described in more detail elsewhere [20], [22]. In brief, following electrophoresis (200 V, 10 min) on agarose gel in 1x TAE buffer (1 mM EDTA, 40 mM Tris-acetate) and UV visualization, 5 µl of the original PCR products were precipitated with 20% polyethylene glycol–2.5 M NaCl [60] and nucleotide sequencing PCRs (2 µl of DNA, 6.98 µl water, 1.0 µl 5x buffer, 0.02 µl BigDye Terminator v3.1 mix [Applied Biosystems, UK] and 0.1 µM of primer) were performed in both directions with cycling parameters as follows: 30 cycles of 96°C for 10 s, 50°C for 5 s, and 60°C for 2 min. Sequencing PCR products were precipitated, cleaned with 95% ethanol, and analyzed with an ABI Prism 3730 automated DNA sequencer (Applied Biosystems, UK). Forward and reverse sequences were assembled using the Staden suite of computer programs [61] and allelic orthologs were assigned numbers giving a 7-locus sequence type (ST). Contemporaneous survey data were augmented with data from published studies [13], [17], [22], [26], [27], [28], [29] obtained from the publicly accessible MLST database (http://pubmlst.org), hosted by the University of Oxford [62].

### Overview of population genetic analysis

A variety of analytical approaches were used to describe the evolutionary history of *C. coli*. Ancestral relatedness of genotypes was assessed using CLONALFRAME [63] to construct genealogies for inference of the *C. coli* phylogeny. Lineage clonality was inferred by investigating congruence of maximum likelihood trees [64]. The timescale of *C. coli* evolution was calibrated using the phylogenetic inference package BEAST [65] and previous estimates of the evolutionary rate in the *Campylobacter* genus based on longitudinal sequences sampled within *C. jejuni* [32]. Source attribution of clinical genotypes was determined using the Asymmetric Island (AI) probabilistic genetic attribution model [21].

### Ancestral relatedness

The genealogy of the *C. coli* STs was estimated using a model-based approach for determining bacterial microevolution: CLONALFRAME [63]. Using this model, clonal relationships are calculated with improved accuracy compared with standard phylogenetic inference techniques for recombining bacteria because the two major sources of allelic polymorphisms (point mutation and recombination) are distinguished. This model has been used successfully to distinguish clades within *C. coli* [25]. Analysis was carried out on all the STs from clinical and non clinical sources. In each case, 7-locus STs were concatenated and the program run with a burn-in of 50,000 iterations followed by 50,000 iterations of sampling. The consensus tree represents combined data from three independent runs with 75% consensus required for inference of relatedness.

### Quantifying clonality

The phylogenetic history of bacteria, evolving according to a clonal model, will be the same for all loci within a genome if it represents a single linkage group irrespective of location. The degree of clonality, therefore, can be estimated by measuring the degree of congruence between phlyogenetic trees constructed for different loci from a single genome. This approach has been employed to compare maximum likelihood (ML) trees describing multiple loci from the genome of, for example, *Borrelia burgdorferi* [64], *Neisseria meningitidis* [66] and *Streptococcus uberis* [67]. Twenty-six genotypes were selected to produce ML trees as in previous studies [66]. Single locus ML trees were constructed for each locus of sample groups containing *C. jejuni, C. jejuni* and *C. coli*, *C. coli* (12 STs from each clade), *C. coli* clade 1, *C. coli* clade 2 and *C. coli* clade 3 and the congruence between trees was determined using the Shimodaira-Hasegawa (SH) test [66], [68]. The difference in congruence log-likelihood (Δ-ln L) of the tree topologies was determined and compared for the 7 ML trees. If evolution is entirely clonal then there should be no significant difference in phylogenetic congruence. The extent of congruence was tested further using the randomized test [66], [68] by comparing the log likelihood for the 7 individual locus ML tree topologies with equivalent values for 200 randomly generated trees of the same size for each gene. If there is more congruence among the ML trees than expected by chance alone then the log likelihood values will fall outside the range calculated for random trees. These analyses were performed using PAUP* version 4 [69].

### The timescale of *C. coli* evolution

The phylogenetic history of *C. coli* was reconstructed, in the context of other species within the genus *Campylobacter*, for which similar MLST schemes have been developed [12], [14], [70]. As described previously [32], 4 of the loci (*glnA*, *glyA*, *tkt* and *uncA*) used in 7-locus STs are common to MLST schemes for all species. STs for each *Campylobacter* species, *C. fetus* (ST-4), *C. helveticus* (ST-2), *C. insulaenigrae* (ST-12), *C. lari* (ST-6), *C. upsaliensis* (ST-25) and 3 STs from *C. jejuni* (ST-21, ST-257 and ST-2678) and *C. coli* clades 1 (ST-1132, ST-3177, ST-1245), 2 (ST-3323, ST-3828, ST-3314) and 3 (ST-3310, ST-3309, ST-2681) were tested to confirm the absence of interspecies recombination between selected STs using a permutation test based on the correlation between physical distance and linkage disequilibrium (LD) [71]. These STs were analysed using the Bayesian phylogenetic package BEAST [65], a codon substitution model [72] and the Yule model of speciation rates [36]. On the timescale of *Campylobacter* evolution all of the STs from isolates in this study were effectively sampled at the same time, therefore there was no data for estimating the rate of evolutionary change. To account for this, we utilized informative prior distributions on the evolutionary parameters comprising the transition-transversion ratio, the dN/dS ratio and the synonymous mutation rate. The priors were taken from the parameters inferred from an analysis of a longitudinal sample of *C. jejuni* collected over a 3-year period [32] assuming a constant rate of evolution within the genus *Campylobacter*.

### Source attribution of clinical genotypes

The Asymmetric Island (AI) probabilistic genetic attribution model [21], was used to characterize the population structure from the genetic data and assign individual isolates in the test set of human isolates independently to source using the training data set. This technique has been used previously [21], [22] and the limitations on the attribution accuracy achievable from a 7-locus profile have been validated by calculating the probability of correct ‘self-assignment’ of a randomly selected sub-set of each host species to the correct origin population [22]. The AI program was run with 1,000 iterations of burn-in followed by 10,000 iterations of sampling, for probabilistic assignment. The putative source of 7-locus genotypes from clinical *C. coli* isolates (500) was assigned by comparison to datasets comprising genotype data from contemporaneous host and environmental/food isolates and genotype data from published sources [13], [17], [22], [26], [27], [28], [29].

## Supporting Information

Table S1 Summary of isolates by ST and source.
(DOC)

Table S2 Allelic diversity at MLST loci of isolates from clinical and non-clinical datasets.
(DOC)

## References

[1] Friedman CJ, Neiman J, Wegener HC, Tauxe RV, Nachamkin I, Blaser MJ (2000). Epidemiology of *Campylobacter jejuni* infections in the United States and other industrialised nations.. *Campylobacter*.

[2] Olson CK, Ethelberg S, van Pelt W, Tauxe RV, Nachamkin I, Szymanski CM, Blaser MJ (2008). Epidemiology of *Campylobacter jejuni* infections in industrialized nations.. *Campylobacter*. 3 ed.

[3] Pebody RG, Ryan MJ, Wall PG (1997). Outbreaks of campylobacter infection: rare events for a common pathogen.. Communicable Disease Reports CDR Reviews.

[4] Kapperud G, Espeland G, Wahl E, Walde A, Herikstad H (2003). Factors associated with increased and decreased risk of *Campylobacter* infection: a prospective case-control study in Norway.. Am J Epidemiol.

[5] Friedman CR, Hoekstra RM, Samuel M, Marcus R, Bender J (2004). Risk factors for sporadic *Campylobacter* infection in the United States: A case-control study in FoodNet sites.. Clinical Infectious Disases.

[6] Allos B (2001). *Campylobacter jejuni* infections: update on emerging issues and trends.. Clinical Infectious Diseases 2001:.

[7] Kessel AS, Gillespie IA, O'Brien SJ, Adak GK, Humphrey TJ (2001). General outbreaks of infectious intestinal disease linked with poultry, England and Wales, 1992-1999.. Commun Dis Public Health.

[8] Tam CC, O'Brien SJ, Adak GK, Meakins SM, Frost JA (2003). *Campylobacter coli* - an important foodborne pathogen.. J Infect.

[9] Gillespie IA, O'Brien SJ, Frost JA, Adak GK, Horby P (2002). A case-case comparison of *Campylobacter coli* and *Campylobacter jejuni* infection: a tool for generating hypotheses.. Emerging Infectious Diseases.

[10] Humphrey T, O'Brien S, Madsen M (2007). Campylobacters as zoonotic pathogens: a food production perspective.. International Journal of Food Microbiology.

[11] Buzby JC, Roberts T (1997). Economic costs and trade impacts of microbial foodbourne illness.. World Health Stat Q.

[12] Dingle KE, Colles FM, Wareing DRA, Ure R, Fox AJ (2001). Multilocus sequence typing system for *Campylobacter jejuni*.. Journal of Clinical Microbiology.

[13] Dingle KE, Colles FM, Falush D, Maiden MC (2005). Sequence typing and comparison of population biology of *Campylobacter coli* and *Campylobacter jejuni*.. Journal of Clinical Microbiology.

[14] Miller WG, On SL, Wang G, Fontanoz S, Lastovica AJ (2005). Extended multilocus sequence typing system for *Campylobacter coli*, *C. lari*, *C. upsaliensis*, and *C. helveticus*.. Journal of Clinical Microbiology.

[15] Maiden MC (2006). Multilocus Sequence Typing of Bacteria.. Annual Review of Microbiology.

[16] Duim B, Wassenaar TM, Rigter A, Wagenaar J (1999). High-resolution genotyping of *Campylobacter* strains isolated from poultry and humans with amplified fragment length polymorphism fingerprinting.. Applied and Environmental Microbiology.

[17] Miller WG, Englen MD, Kathariou S, Wesley IV, Wang G (2006). Identification of host-associated alleles by multilocus sequence typing of *Campylobacter coli* strains from food animals.. Microbiology.

[18] Sheppard SK, Colles F, Richardson J, Cody AJ, Elson R (2010). Host Association of Campylobacter Genotypes Transcends Geographic Variation.. Applied and Environmental Microbiology.

[19] Rotariu O, Dallas JF, Ogden ID, MacRae M, Sheppard SK (2009). Spatiotemporal homogeneity of Campylobacter subtypes from cattle and sheep across northeastern and southwestern Scotland.. Appl Environ Microbiol.

[20] Sheppard SK, Dallas JF, MacRae M, McCarthy ND, Sproston EL (2009). Campylobacter genotypes from food animals, environmental sources and clinical disease in Scotland 2005/6.. International Journal of Food Microbiology.

[21] Wilson DJ, Gabriel E, Leatherbarrow AJH, Cheesbrough J, Gee S (2008). Tracing the source of campylobacteriosis.. PLoS Genetics.

[22] Sheppard SK, Dallas JF, Strachan NJ, MacRae M, McCarthy ND (2009). Campylobacter genotyping to determine the source of human infection.. Clinical Infectious Diseases.

[23] Mullner P, Jones G, Noble A, Spencer SE, Hathaway S (2009). Source attribution of food-borne zoonoses in New Zealand: a modified Hald model.. Risk Anal.

[24] Strachan NJ, Gormley FJ, Rotariu O, Ogden ID, Miller G (2009). Attribution of Campylobacter infections in northeast Scotland to specific sources by use of multilocus sequence typing.. Journal of Infectious Diseases.

[25] Sheppard SK, McCarthy ND, Falush D, Maiden MC (2008). Convergence of *Campylobacter* species: implications for bacterial evolution.. Science.

[26] Sopwith W, Birtles A, Matthews M, Fox A, Gee S (2009). Investigation of food and environmental exposures relating to epidemiology of *Campylobacter coli* in humans in North West England.. Applied and Environmental Microbiology.

[27] Thakur S, Gebreyes WA (2005). *Campylobacter coli* in swine production: antimicrobial resistance mechanisms and molecular epidemiology.. Journal of Clinical Microbiology.

[28] Kinana AD, Cardinale E, Bahsoun I, Tall F, Sire JM (2007). *Campylobacter coli* isolates derived from chickens in Senegal: Diversity, genetic exchange with *Campylobacter jejuni* and quinolone resistance.. Research in Microbiology.

[29] Litrup E, Torpdahl M, Nielsen EM (2007). Multilocus sequence typing performed on *Campylobacter coli* isolates from humans, broilers, pigs and cattle originating in Denmark.. Journal of Applied Microbiology.

[30] McCarthy ND, Colles FM, Dingle KE, Bagnall MC, Manning G (2007). Host-associated genetic import in *Campylobacter jejuni*.. Emerging Infectious Diseases.

[31] Fearnhead P (2008). Computational methods for complex stochastic systems: a review of some alternatives to MCMC.. Statistics and Computing.

[32] Wilson DJ, Gabriel E, Leatherbarrow AJ, Cheesbrough J, Gee S (2009). Rapid evolution and the importance of recombination to the gastroenteric pathogen Campylobacter jejuni.. Mol Biol Evol.

[33] Achtman M, Morelli G, Zhu P, Wirth T, Diehl I (2004). Microevolution and history of the plague bacillus, *Yersinia pestis*.. Proceedings of the National Academy of Sciences USA.

[34] Roumagnac P, Weill FX, Dolecek C, Baker S, Brisse S (2006). Evolutionary history of *Salmonella typhi*.. Science.

[35] Ochman H, Wilson AC (1987). Evolution in bacteria: evidence for a universal substitution rate in cellular genomes.. Journal of Molecular Evolution.

[36] Yule GU (1924). A mathematical theory of evolution, based on the conclusions of DR. J.C. Willis.. Philosophical Transactions of the Royal Society of London Series B: Biological Sciences.

[37] Khanna MR, Bhavsar SP, Kapadnis BP (2006). Effect of temperature on growth and chemotactic behaviour of *Campylobacter jejuni*.. Letters in Applied Microbiology.

[38] Konkel ME, Christensen JE, Dhillon AS, Lane AB, Hare-Sanford R (2007). *Campylobacter jejuni* strains compete for colonization in broiler chicks.. Applied and Environmental Microbiology.

[39] Drake JW (1991). A constant rate of spontaneous mutation in DNA-based microbes.. Proceedings of the National Academy of Sciences U S A.

[40] Fraser C, Hanage WP, Spratt BG (2007). Recombination and the nature of bacterial speciation.. Science.

[41] Eggleston AK, West SC (1997). Recombination initiation: easy as A, B, C, D… chi?. Current Biology.

[42] Zhu P, van der Ende A, Falush D, Brieske N, Morelli G (2001). Fit genotypes and escape variants of subgroup III *Neisseria meningitidis* during three pandemics of epidemic meningitis.. Proceedings of the National Academy of Sciences USA.

[43] Achtman M, Wagner M (2008). Microbial diversity and the genetic nature of microbial species.. Nat Rev Microbiol.

[44] Strachan NJ, Gormley FJ, Rotariu O, Ogden ID, Miller G (2008). Attribution of Campylobacter infections in northeast Scotland to specific sources by use of multilocus sequence typing.. Journal of Infectious Diseases.

[45] Moran NA (2003). Tracing the evolution of gene loss in obligate bacterial symbionts.. Curr Opin Microbiol.

[46] Zhang W, Qi W, Albert TJ, Motiwala AS, Alland D (2006). Probing genomic diversity and evolution of Escherichia coli O157 by single nucleotide polymorphisms.. Genome Res.

[47] Pupo GM, Lan R, Reeves PR (2000). Multiple independent origins of Shigella clones of *Escherichia coli* and convergent evolution of many of their characteristics.. Proceedings of the National Academy of Sciences USA.

[48] Lenski RE, Winkworth CL, Riley MA (2003). Rates of DNA sequence evolution in experimental populations of *Escherichia coli* during 20,000 generations.. Journal of Molecular Evolution.

[49] Ochman H (2003). Neutral mutations and neutral substitutions in bacterial genomes.. Molecular Biology and Evolution.

[50] Ammerman AJ, Cavalii-Sforza LL (1984). The neolithic transition and the genetics of populations in Europe.

[51] McCorriston J, Hole F (1991). The ecology of seasonal stress and the origin of agriculture in the Near East.. American Anthropologist.

[52] Zvelebil M, Dolukhanov P (1991). The transition to farming in eastern and northern europe.. Journal of World Prehistory.

[53] Falush D, Kraft C, Taylor NS, Correa P, Fox JG (2001). Recombination and mutation during long-term gastric colonization by *Helicobacter pylori*: estimates of clock rates, recombination size, and minimal age.. Proceedings of the National Academy of Sciences USA.

[54] Perez-Losada M, Crandall KA, Zenilman J, Viscidi RP (2007). Temporal trends in gonococcal population genetics in a high prevalence urban community.. Infect Genet Evol.

[55] Feng L, Reeves PR, Lan R, Ren Y, Gao C (2008). A recalibrated molecular clock and independent origins for the cholera pandemic clones.. PLoS One.

[56] Lowder BV, Guinane CM, Ben Zakour NL, Weinert LA, Conway-Morris A (2009). Recent human-to-poultry host jump, adaptation, and pandemic spread of Staphylococcus aureus.. proceedings of the National Academy of Sciences U S A.

[57] Debruyne L, Gevers D, Vandamme P, Nachamkin I, Szymanski CM, Blaser MJ (2008). Taxonomy of the family *Campylobacteraceae*.. *Campylobacter*. 3rd ed.

[58] Gormley FJ, Macrae M, Forbes KJ, Ogden ID, Dallas JF (2008). Has retail chicken played a role in the decline of human campylobacteriosis?. Appl Environ Microbiol.

[59] Humphrey TJ, Jorgensen F, Frost JA, Wadda H, Domingue G (2005). Prevalence and subtypes of ciprofloxacin-resistant Campylobacter spp. in commercial poultry flocks before, during, and after treatment with fluoroquinolones.. Antimicrob Agents Chemother.

[60] Embley TM (1991). The linear PCR reaction: a simple and robust method for sequencing amplified rRNA genes.. Letters in Applied Microbiology.

[61] Staden R (1996). The Staden sequence analysis package.. Molecular Biotechnology.

[62] Jolley KA, Chan MS, Maiden MC (2004). mlstdbNet - distributed multi-locus sequence typing (MLST) databases.. BMC Bioinformatics.

[63] Didelot X, Falush D (2007). Inference of bacterial microevolution using multilocus sequence data.. Genetics.

[64] Dykhuizen DE, Polin DS, Dunn JJ, Wilske B, Preac Mursic V (1993). *Borrelia burgdorferi* is clonal: implications for taxonomy and vaccine development.. Proceedings of the National Academy of Sciences USA.

[65] Drummond AJ, Nicholls GK, Rodrigo AG, Solomon W (2002). Estimating mutation parameters, population history and genealogy simultaneously from temporally spaced sequence data.. Genetics.

[66] Holmes EC, Urwin R, Maiden MCJ (1999). The influence of recombination on the population structure and evolution of the human pathogen *Neisseria meningitidis*.. Molecular Biology and Evolution.

[67] Coffey TJ, Pullinger GD, Urwin R, Jolley KA, Wilson SM (2006). First insights into the evolution of *Streptococcus uberis*: a multilocus sequence typing scheme that enables investigation of its population biology.. Applied and Environmental Microbiology.

[68] Feil EJ, Holmes EC, Bessen DE, Chan MS, Day NP (2001). Recombination within natural populations of pathogenic bacteria: Short- term empirical estimates and long-term phylogenetic consequences.. Proceedings of the National Academy of Sciences USA.

[69] Swofford D (1998). PAUP* Phylogenetic analysis using parsimony and other methods..

[70] van Bergen MA, Dingle KE, Maiden MC, Newell DG, van der Graaf-Van Bloois L (2005). Clonal nature of *Campylobacter fetus* as defined by multilocus sequence typing.. Journal of Clinical Microbiology.

[71] McVean G, Awadalla P, Fearnhead P (2002). A coalescent-based method for detecting and estimating recombination from gene sequences.. Genetics.

[72] Nielson R, Yang Z (1998). Likelihood models for detecting positively selected amino acid sites and applications to the HIV-1 envelope gene.. Genetics.

