# Supplementary material for: Evolution of an Agriculture-Associated Disease Causing Campylobacter coli Clade: Evidence from National Surveillance Data in Scotland
Source: PLoS One. 2010 Dec 15;5(12):e15708. doi: 10.1371/journal.pone.0015708 (PMC3002284; doi:10.1371/journal.pone.0015708)
Supplement: Table S1 — Summary of isolates by ST and source. (DOC) [file pone.0015708.s001.doc]

Table S1. Summary of isolates by ST and source.

| ST | Clade^1^ | Source of isolates | | | | | | |
| --- | --- | --- | --- | --- | --- | --- | --- | --- |
|  |  | Cattle | Chicken | Clinical | Riparian environment^2^ | Pig | Sheep | Turkey |
| 825 | 1 | 1 | 42 | 48 |  | 4 | 20 |  |
| 827 | 1 | 16 | 43 | 174 |  | 1 | 21 |  |
| 828 | 1 | 4 | 8 | 13 |  | 4 |  |  |
| 829 | 1 |  | 51 | 18 |  | 1 |  |  |
| 830 | 1 |  | 2 | 4 |  | 1 |  |  |
| 831 | 1 |  |  | 1 |  |  |  |  |
| 832 | 1 |  | 3 | 4 |  |  |  |  |
| 854 | 1 |  | 4 | 3 |  | 19 | 1 |  |
| 855 | 1 |  | 28 | 22 |  |  |  |  |
| 860 | 1 |  | 2 | 4 |  | 1 |  |  |
| 867 | 1 |  | 10 | 1 |  |  |  |  |
| 872 | 1 | 2 | 2 | 17 |  |  |  |  |
| 887 | 1 |  | 1 | 1 |  | 1 |  |  |
| 888 | 1 |  |  |  |  | 3 |  |  |
| 889 | 1 |  |  |  |  |  |  | 6 |
| 890 | 1 |  | 1 | 1 |  | 9 |  |  |
| 894 | 1 |  | 6 |  |  |  |  |  |
| 898 | 1 |  | 1 |  |  |  |  |  |
| 899 | 1 | 1 | 3 |  |  | 2 |  |  |
| 901 | 1 |  |  | 1 |  |  |  |  |
| 902 | 1 |  | 2 | 3 |  |  |  |  |
| 962 | 1 | 3 | 6 | 17 |  |  | 9 |  |
| 1009 | 1 |  | 10 | 1 |  |  |  |  |
| 1016 | 1 |  |  | 2 |  |  |  |  |
| 1017 | 1 |  | 28 |  |  |  |  |  |
| 1018 | 1 |  | 3 | 1 |  |  |  |  |
| 1050 | 1 |  | 3 |  |  |  |  |  |
| 1054 | 1 |  |  |  |  | 1 |  |  |
| 1055 | 1 |  | 4 | 4 |  | 3 |  |  |
| 1056 | 1 | 1 | 1 |  |  | 4 |  |  |
| 1058 | 1 |  | 3 | 2 |  | 3 |  |  |
| 1059 | 1 |  |  | 1 |  | 3 |  |  |
| 1061 | 1 | 1 | 1 |  |  |  |  |  |
| 1063 | 1 |  | 2 |  |  |  |  |  |
| 1065 | 1 |  | 1 |  |  |  |  |  |
| 1068 | 1 | 52 |  |  |  | 13 |  |  |
| 1082 | 1 |  | 4 |  |  |  |  |  |
| 1088 | 1 |  | 1 |  |  |  |  |  |
| 1090 | 1 |  | 3 |  |  |  |  |  |
| 1092 | 1 |  |  |  |  |  |  | 1 |
| 1093  Table S1 - continued. | 1 |  |  |  |  | 1 |  |  |
| 1094 | 1 |  |  |  |  | 3 |  |  |
| 1095 | 1 | 1 |  |  |  | 1 |  |  |
| 1096 | 1 |  | 1 | 3 |  | 12 |  |  |
| 1097 | 1 |  |  |  |  | 2 |  |  |
| 1098 | 1 |  |  |  |  | 1 |  |  |
| 1099 | 1 |  |  |  |  | 9 |  |  |
| 1100 | 1 |  |  |  |  | 1 |  |  |
| 1101 | 1 |  |  |  |  |  |  | 22 |
| 1102 | 1 |  |  |  |  | 5 |  |  |
| 1103 | 1 |  |  |  |  | 1 |  |  |
| 1104 | 1 | 1 |  |  |  | 5 |  |  |
| 1105 | 1 |  |  |  |  | 5 |  |  |
| 1106 | 1 |  |  |  |  | 9 |  |  |
| 1107 | 1 |  |  |  |  | 1 |  |  |
| 1108 | 1 |  |  |  |  | 1 |  |  |
| 1109 | 1 |  | 2 | 1 |  | 4 |  |  |
| 1110 | 1 |  |  |  |  |  |  | 1 |
| 1111 | 1 |  |  |  |  | 1 |  |  |
| 1112 | 1 |  |  |  |  | 6 |  |  |
| 1113 | 1 |  | 1 |  |  | 5 |  |  |
| 1114 | 1 |  |  |  |  | 2 |  |  |
| 1115 | 1 |  |  |  |  | 4 |  |  |
| 1116 | 1 |  |  |  |  | 5 |  |  |
| 1117 | 1 |  |  |  |  | 2 |  |  |
| 1118 | 1 |  |  |  |  | 1 |  |  |
| 1119 | 1 |  | 4 |  |  |  |  | 1 |
| 1120 | 1 |  | 1 |  |  |  |  |  |
| 1121 | 1 |  | 2 |  |  |  |  | 8 |
| 1122 | 1 |  |  |  |  | 1 |  |  |
| 1123 | 1 |  |  |  |  | 8 |  |  |
| 1124 | 1 |  |  |  |  | 4 |  |  |
| 1125 | 1 |  |  |  |  | 2 |  |  |
| 1126 | 1 |  |  |  |  |  |  | 13 |
| 1127 | 1 |  |  |  |  | 1 |  |  |
| 1128 | 1 |  |  |  |  | 1 |  |  |
| 1129 | 1 |  | 1 |  |  |  |  |  |
| 1130 | 1 |  | 1 |  |  | 5 |  |  |
| 1131 | 1 |  |  |  |  | 1 |  |  |
| 1132 | 1 |  |  |  |  | 2 |  |  |
| 1133 | 1 |  |  |  |  | 1 |  |  |
| 1134 | 1 |  |  |  |  | 6 |  |  |
| 1135 | 1 |  |  |  |  |  |  | 8 |
| 1136 | 1 |  |  |  |  |  |  | 1 |
| 1137 | 1 |  |  |  |  |  |  | 9 |
| 1138 | 1 |  |  |  |  |  |  | 1 |
| 1139  Table S1 - continued. | 1 |  |  |  |  | 1 |  |  |
| 1140 | 1 |  |  |  |  | 1 |  |  |
| 1141 | 1 |  |  |  |  | 1 |  |  |
| 1142 | 1 |  | 3 |  |  | 11 |  |  |
| 1143 | 1 |  |  |  |  | 7 |  |  |
| 1144 | 1 |  |  |  |  | 1 |  |  |
| 1145 | 1 |  |  |  |  | 1 |  |  |
| 1146 | 1 |  |  |  |  | 1 |  |  |
| 1147 | 1 |  |  |  |  | 1 |  |  |
| 1148 | 1 |  | 1 |  |  |  |  |  |
| 1149 | 1 |  |  |  |  |  |  | 3 |
| 1150 | 1 |  |  |  |  |  |  | 12 |
| 1151 | 1 |  |  |  |  | 3 |  |  |
| 1152 | 1 |  |  |  |  | 1 |  |  |
| 1153 | 1 |  |  |  |  | 2 |  |  |
| 1154 | 1 |  |  |  |  |  |  | 3 |
| 1155 | 1 |  |  |  |  |  |  | 1 |
| 1156 | 1 |  | 1 |  |  |  |  |  |
| 1157 | 1 |  |  |  |  | 3 |  |  |
| 1158 | 1 |  |  |  |  | 1 |  |  |
| 1159 | 1 |  |  |  |  | 2 |  |  |
| 1160 | 1 |  |  |  |  |  |  | 1 |
| 1161 | 1 |  |  |  |  |  |  | 7 |
| 1163 | 1 |  |  |  |  |  |  | 1 |
| 1164 | 1 |  |  |  |  | 1 |  |  |
| 1165 | 1 |  |  |  |  | 4 |  |  |
| 1166 | 1 |  | 1 | 1 |  |  |  |  |
| 1167 | 1 |  | 1 |  |  |  |  |  |
| 1168 | 1 |  | 1 |  |  |  |  |  |
| 1169 | 1 |  | 4 |  |  |  |  |  |
| 1170 | 1 |  | 3 |  |  |  |  | 1 |
| 1171 | 1 |  |  |  |  |  |  | 1 |
| 1172 | 1 |  |  |  |  | 1 |  |  |
| 1173 | 1 |  | 4 | 2 |  |  | 1 |  |
| 1174 | 1 |  | 1 |  |  |  |  |  |
| 1175 | 1 |  |  |  |  |  |  | 1 |
| 1176 | 1 |  | 1 |  |  |  |  |  |
| 1177 | 1 |  |  |  |  | 2 |  |  |
| 1178 | 1 |  |  |  |  | 1 |  |  |
| 1179 | 1 |  | 1 |  |  |  |  |  |
| 1180 | 1 |  | 1 |  |  |  |  |  |
| 1181 | 1 |  | 5 | 2 |  |  |  |  |
| 1182 | 1 |  | 1 |  |  |  |  |  |
| 1183 | 1 |  | 1 |  |  |  |  |  |
| 1184 | 1 |  |  |  |  |  |  | 1 |
| 1185 | 1 |  |  |  |  | 1 |  |  |
| 1186  Table S1 - continued. | 1 |  |  |  |  | 1 |  |  |
| 1187 | 1 |  |  |  |  | 1 |  |  |
| 1188 | 1 |  |  |  |  |  |  | 1 |
| 1189 | 1 |  |  |  |  | 1 |  |  |
| 1190 | 1 |  | 1 |  |  |  |  |  |
| 1191 | 1 |  | 1 | 2 |  |  |  |  |
| 1192 | 1 |  |  |  |  |  |  | 1 |
| 1193 | 1 |  |  |  |  |  |  | 1 |
| 1194 | 1 |  |  |  |  |  |  | 1 |
| 1195 | 1 |  | 1 |  |  |  |  |  |
| 1196 | 1 |  |  |  |  | 1 |  |  |
| 1197 | 1 |  |  |  |  | 1 |  |  |
| 1198 | 1 |  |  |  |  |  |  | 1 |
| 1199 | 1 |  |  |  |  |  |  | 1 |
| 1200 | 1 |  |  |  |  | 2 |  |  |
| 1201 | 1 |  |  |  |  | 1 |  |  |
| 1202 | 1 |  |  |  |  | 1 |  |  |
| 1203 | 1 |  | 1 |  |  | 1 |  |  |
| 1204 | 1 |  |  |  |  | 1 |  |  |
| 1205 | 1 | 1 |  |  |  |  |  |  |
| 1238 | 1 |  |  |  |  | 1 |  |  |
| 1239 | 1 |  | 1 |  |  |  |  |  |
| 1240 | 1 |  |  |  |  | 1 |  |  |
| 1241 | 1 |  | 1 |  |  |  |  |  |
| 1242 | 1 |  |  |  |  |  |  | 1 |
| 1243 | 1 | 1 |  | 1 |  |  |  |  |
| 1245 | 1 | 1 |  |  |  |  |  |  |
| 1246 | 1 |  |  |  |  | 2 |  |  |
| 1247 | 1 |  | 1 |  |  |  |  |  |
| 1248 | 1 |  |  |  |  | 2 |  |  |
| 1405 | 1 |  | 4 | 3 |  |  |  |  |
| 1413 | 1 |  |  |  |  | 7 |  |  |
| 1414 | 1 |  |  |  |  | 2 |  |  |
| 1415 | 1 |  |  |  |  | 1 |  |  |
| 1416 | 1 |  |  |  |  | 3 |  |  |
| 1417 | 1 |  | 1 |  |  | 2 |  |  |
| 1418 | 1 |  |  |  |  | 1 |  |  |
| 1419 | 1 |  |  |  |  | 1 |  |  |
| 1420 | 1 |  |  |  |  | 1 |  |  |
| 1421 | 1 |  |  |  |  | 1 |  |  |
| 1422 | 1 |  |  |  |  | 1 |  |  |
| 1423 | 1 |  |  |  |  | 1 |  |  |
| 1424 | 1 |  |  |  |  | 1 |  |  |
| 1425 | 1 |  |  |  |  | 2 |  |  |
| 1426 | 1 |  |  |  |  | 1 |  |  |
| 1427 | 1 |  | 3 | 1 |  | 1 |  |  |
| 1428  Table S1 - continued. | 1 |  |  |  |  | 1 |  |  |
| 1429 | 1 |  |  |  |  | 1 |  |  |
| 1430 | 1 |  |  |  |  | 1 |  |  |
| 1431 | 1 |  |  |  |  | 1 |  |  |
| 1432 | 1 |  |  |  |  | 3 |  |  |
| 1433 | 1 |  |  |  |  | 1 |  |  |
| 1434 | 1 |  |  |  |  | 1 |  |  |
| 1435 | 1 |  |  |  |  | 1 |  |  |
| 1436 | 1 |  |  |  |  | 1 |  |  |
| 1437 | 1 |  |  |  |  | 1 |  |  |
| 1438 | 1 |  | 1 |  |  | 2 |  |  |
| 1439 | 1 |  |  |  |  | 2 |  |  |
| 1440 | 1 |  |  |  |  | 1 |  |  |
| 1441 | 1 |  |  |  |  | 1 |  |  |
| 1442 | 1 |  |  |  |  | 1 |  |  |
| 1443 | 1 |  |  |  |  | 1 |  |  |
| 1445 | 1 |  |  |  |  | 1 |  |  |
| 1446 | 1 |  |  |  |  | 2 |  |  |
| 1447 | 1 |  |  |  |  | 1 |  |  |
| 1448 | 1 |  |  |  |  | 1 |  |  |
| 1449 | 1 |  |  |  |  | 1 |  |  |
| 1450 | 1 |  |  | 1 |  | 6 |  |  |
| 1451 | 1 |  |  |  |  | 1 |  |  |
| 1452 | 1 |  |  |  |  | 2 |  |  |
| 1453 | 1 |  |  |  |  | 1 |  |  |
| 1454 | 1 |  |  |  |  | 1 |  |  |
| 1455 | 1 |  |  |  |  | 1 |  |  |
| 1464 | 1 |  |  |  |  | 3 |  |  |
| 1465 | 1 |  |  |  |  | 1 |  |  |
| 1466 | 1 |  |  |  |  | 1 |  |  |
| 1469 | 1 |  |  |  |  | 1 |  |  |
| 1493 | 1 |  | 5 |  |  |  |  |  |
| 1494 | 1 |  | 5 |  |  |  |  |  |
| 1531 | 1 |  |  | 1 |  |  |  |  |
| 1541 | 1 |  | 3 | 3 |  |  |  |  |
| 1543 | 1 |  |  | 1 |  | 1 |  |  |
| 1544 | 1 | 1 | 4 |  |  | 3 |  |  |
| 1545 | 1 |  | 2 |  |  | 4 |  |  |
| 1546 | 1 |  |  |  |  | 1 |  |  |
| 1547 | 1 |  |  |  |  | 1 |  |  |
| 1548 | 1 |  |  |  |  | 1 |  |  |
| 1549 | 1 |  |  |  |  | 3 |  |  |
| 1550 | 1 |  |  |  |  | 1 |  |  |
| 1551 | 1 |  |  |  |  | 2 |  |  |
| 1552 | 1 |  |  |  |  | 1 |  |  |
| 1553 | 1 |  |  |  |  | 1 |  |  |
| 1554  Table S1 - continued. | 1 |  |  |  |  | 1 |  |  |
| 1555 | 1 |  |  |  |  | 1 |  |  |
| 1556 | 1 |  |  |  |  | 2 |  |  |
| 1557 | 1 |  |  |  |  | 1 |  |  |
| 1558 | 1 |  |  |  |  | 3 |  |  |
| 1559 | 1 |  |  |  |  | 1 |  |  |
| 1560 | 1 |  |  |  |  | 1 |  |  |
| 1561 | 1 |  | 2 |  |  | 1 |  |  |
| 1562 | 1 |  |  |  |  | 2 |  |  |
| 1563 | 1 |  | 3 | 1 |  | 1 |  |  |
| 1564 | 1 |  | 1 |  |  |  |  |  |
| 1565 | 1 |  |  |  |  | 1 |  |  |
| 1566 | 1 |  |  |  |  | 1 |  |  |
| 1567 | 1 |  | 1 |  |  |  |  |  |
| 1568 | 1 |  |  |  |  | 1 |  |  |
| 1569 | 1 |  | 1 |  |  |  |  |  |
| 1570 | 1 |  | 1 |  |  |  |  |  |
| 1571 | 1 |  | 1 |  |  |  |  |  |
| 1572 | 1 |  | 2 |  |  |  |  |  |
| 1573 | 1 |  | 1 |  |  |  |  |  |
| 1574 | 1 |  | 1 |  |  |  |  |  |
| 1575 | 1 |  | 1 | 3 |  |  |  |  |
| 1576 | 1 |  | 1 |  |  |  |  |  |
| 1578 | 1 | 1 | 1 |  |  |  |  |  |
| 1579 | 1 |  | 1 |  |  |  |  |  |
| 1580 | 1 |  | 2 |  |  |  |  |  |
| 1585 | 1 |  | 3 |  |  |  |  |  |
| 1586 | 1 |  |  | 4 |  |  |  |  |
| 1597 | 1 |  | 5 |  |  |  |  |  |
| 1600 | 1 |  | 9 |  |  |  |  |  |
| 1601 | 1 |  | 2 |  |  |  |  |  |
| 1602 | 1 |  | 1 |  |  |  |  |  |
| 1603 | 1 |  | 2 |  |  |  |  |  |
| 1604 | 1 |  | 3 |  |  |  |  |  |
| 1605 | 1 |  | 1 |  |  |  |  |  |
| 1610 | 1 |  | 1 |  |  |  |  |  |
| 1611 | 1 |  | 1 |  |  |  |  |  |
| 1614 | 1 |  | 17 | 18 |  |  | 2 |  |
| 1617 | 1 |  |  |  |  | 1 |  |  |
| 1618 | 1 |  |  | 1 |  |  |  |  |
| 1624 | 1 |  |  |  |  | 3 |  |  |
| 1628 | 1 |  | 2 | 1 |  |  |  |  |
| 1675 | 1 | 1 |  |  |  |  |  |  |
| 1676 | 1 | 1 |  |  |  |  |  |  |
| 1680 | 1 |  | 1 |  |  |  |  |  |
| 1681 | 1 |  | 2 | 1 |  |  |  |  |
| 1761  Table S1 - continued. | 1 |  | 5 | 1 |  |  |  |  |
| 1769 | 1 |  |  | 1 |  |  |  |  |
| 1772 | 2 |  |  |  | 1 |  |  |  |
| 1773 | 1 |  | 4 | 9 |  |  |  |  |
| 1774 | 1 |  | 7 | 25 |  |  |  |  |
| 1826 | 1 |  |  |  |  | 1 |  |  |
| 1827 | 1 |  |  |  |  | 1 |  |  |
| 1834 | 1 |  |  |  |  | 1 |  |  |
| 1837 | 1 |  |  | 1 |  |  |  |  |
| 1957 | 1 |  | 1 | 2 |  |  |  |  |
| 1975 | 3 |  |  |  | 1 |  |  |  |
| 1976 | 2 |  |  |  | 1 |  |  |  |
| 1980 | 3 |  |  |  | 1 |  |  |  |
| 1981 | 2 |  |  |  | 1 |  |  |  |
| 1982 | 3 |  |  |  | 1 |  |  |  |
| 1983 | 3 |  |  |  | 1 |  |  |  |
| 1984 | 2 |  |  |  | 1 |  |  |  |
| 1985 | 3 |  |  |  | 1 |  |  |  |
| 1988 | 2 |  |  |  | 1 |  |  |  |
| 1989 | 3 |  |  |  | 1 |  |  |  |
| 1990 | 2 |  |  |  | 1 |  |  |  |
| 1991 | 2 |  |  |  | 1 |  |  |  |
| 1992 | 3 |  |  |  | 2 |  |  |  |
| 1996 | 3 |  |  |  | 1 |  |  |  |
| 1997 | 3 |  |  |  | 1 |  |  |  |
| 1998 | 3 |  |  |  | 1 |  |  |  |
| 1999 | 2 |  |  |  | 1 |  |  |  |
| 2000 | 3 |  |  |  | 1 |  |  |  |
| 2001 | 2 |  |  |  | 1 |  |  |  |
| 2002 | 3 |  |  |  | 1 |  |  |  |
| 2004 | 1 |  | 2 |  |  |  |  |  |
| 2005 | 3 |  |  |  | 1 |  |  |  |
| 2006 | 1 |  |  | 1 |  |  |  |  |
| 2007 | 3 |  |  |  | 1 |  |  |  |
| 2010 | 3 |  |  |  | 1 |  |  |  |
| 2012 | 2 |  |  |  | 1 |  |  |  |
| 2015 | 2 |  |  |  | 3 |  |  |  |
| 2016 | 2 |  |  |  | 2 |  |  |  |
| 2017 | 2 |  |  |  | 1 |  |  |  |
| 2018 | 2 |  |  |  | 1 |  |  |  |
| 2020 | 3 |  |  |  | 1 |  |  |  |
| 2021 | 2 |  |  |  | 1 |  |  |  |
| 2022 | 1 |  | 1 |  |  |  |  |  |
| 2023 | 3 |  |  |  | 1 |  |  |  |
| 2024 | 2 |  |  |  | 1 |  |  |  |
| 2025 | 2 |  |  |  | 1 |  |  |  |
| 2050  Table S1 - continued. | 1 |  |  | 1 |  |  |  |  |
| 2051 | 1 |  |  | 1 |  |  |  |  |
| 2055 | 1 |  |  | 1 |  |  |  |  |
| 2064 | 1 |  |  | 1 |  |  |  |  |
| 2073 | 1 |  |  | 1 |  |  |  |  |
| 2074 | 1 |  |  | 1 |  |  |  |  |
| 2075 | 1 |  |  | 1 |  |  |  |  |
| 2077 | 1 |  |  | 1 |  |  |  |  |
| 2097 | 1 |  |  | 1 |  |  |  |  |
| 2101 | 1 |  |  | 1 |  |  |  |  |
| 2126 | 1 |  |  | 1 |  |  |  |  |
| 2129 | 1 |  |  | 1 |  |  |  |  |
| 2137 | 1 |  |  | 1 |  |  |  |  |
| 2139 | 1 |  |  | 1 |  |  |  |  |
| 2142 | 1 |  |  | 2 |  |  |  |  |
| 2172 | 1 |  |  | 1 |  |  |  |  |
| 2174 | 1 |  |  | 1 |  |  |  |  |
| 2177 | 1 |  |  | 1 |  |  |  |  |
| 2178 | 1 | 1 | 1 | 7 |  |  |  |  |
| 2179 | 1 |  |  | 1 |  |  |  |  |
| 2183 | 1 |  |  | 1 |  |  |  |  |
| 2193 | 1 |  |  | 1 |  |  |  |  |
| 2194 | 1 |  |  | 1 |  |  |  |  |
| 2195 | 1 |  | 4 | 2 |  |  |  |  |
| 2196 | 1 |  |  | 1 |  |  |  |  |
| 2204 | 1 |  |  | 1 |  |  |  |  |
| 2205 | 1 |  |  | 2 |  |  |  |  |
| 2220 | 1 |  |  |  |  | 1 |  |  |
| 2256 | 1 |  | 1 |  |  |  |  |  |
| 2273 | 1 |  | 8 | 1 |  |  |  |  |
| 2298 | 1 |  | 1 | 2 |  |  |  |  |
| 2300 | 1 |  |  | 1 |  |  |  |  |
| 2301 | 1 |  |  | 1 |  |  |  |  |
| 2302 | 1 |  |  | 1 |  |  |  |  |
| 2323 | 1 |  |  | 1 |  |  |  |  |
| 2460 | 1 |  | 2 |  |  |  |  |  |
| 2461 | 1 |  | 2 |  |  |  |  |  |
| 2462 | 1 |  | 1 |  |  |  |  |  |
| 2464 | 1 |  | 1 |  |  |  |  |  |
| 2465 | 1 |  | 2 |  |  |  |  |  |
| 2466 | 1 |  | 2 |  |  |  |  |  |
| 2468 | 1 |  | 1 |  |  |  |  |  |
| 2469 | 1 |  | 2 |  |  |  |  |  |
| 2471 | 1 |  | 1 |  |  |  |  |  |
| 2472 | 1 |  | 1 |  |  |  |  |  |
| 2473 | 1 |  | 1 |  |  |  |  |  |
| 2474  Table S1 - continued. | 1 |  | 1 |  |  |  |  |  |
| 2475 | 1 |  | 1 |  |  |  |  |  |
| 2476 | 1 |  | 1 |  |  |  |  |  |
| 2477 | 1 |  | 1 |  |  |  |  |  |
| 2478 | 1 |  | 1 |  |  |  |  |  |
| 2479 | 1 |  | 1 |  |  |  |  |  |
| 2480 | 1 |  | 1 |  |  |  |  |  |
| 2481 | 1 |  | 1 |  |  |  |  |  |
| 2482 | 1 |  | 1 |  |  |  |  |  |
| 2483 | 1 |  | 1 |  |  |  |  |  |
| 2484 | 1 |  | 1 |  |  |  |  |  |
| 2485 | 3 |  | 1 |  |  |  |  |  |
| 2486 | 1 |  | 1 |  |  |  |  |  |
| 2487 | 1 |  | 1 |  |  |  |  |  |
| 2488 | 1 |  | 1 |  |  |  |  |  |
| 2490 | 1 |  | 1 |  |  |  |  |  |
| 2493 | 1 |  | 1 |  |  |  |  |  |
| 2494 | 1 |  | 1 |  |  |  |  |  |
| 2495 | 1 |  | 1 |  |  |  |  |  |
| 2508 | 1 |  |  |  |  | 1 |  |  |
| 2587 | 1 |  | 4 |  |  |  |  |  |
| 2588 | 1 |  | 2 |  |  |  |  |  |
| 2598 | 1 |  | 1 |  |  |  |  |  |
| 2608 | 1 |  | 1 |  |  |  |  |  |
| 2614 | 1 |  | 1 |  |  |  |  |  |
| 2616 | 1 |  | 1 |  |  |  |  |  |
| 2617 | 1 |  | 1 |  |  |  |  |  |
| 2621 | 1 |  | 1 |  |  |  |  |  |
| 2623 | 1 |  | 1 |  |  |  |  |  |
| 2658 | 1 |  |  |  |  | 1 |  |  |
| 2660 | 1 | 1 |  |  |  |  |  |  |
| 2662 | 1 | 1 |  |  |  |  |  |  |
| 2665 | 1 | 1 |  |  |  |  |  |  |
| 2681 | 3 |  | 1 |  |  |  |  |  |
| 2682 | 1 |  | 1 | 1 |  |  |  |  |
| 2684 | 1 |  | 1 |  |  |  |  |  |
| 2689 | 1 |  | 1 |  |  |  |  |  |
| 2695 | 1 |  |  |  |  | 1 |  |  |
| 2696 | 1 |  |  |  |  | 2 |  |  |
| 2697 | 1 |  |  |  |  | 1 |  |  |
| 2718 | 1 |  |  |  |  | 1 |  |  |
| 2773 | 1 | 1 |  |  |  |  |  |  |
| 2774 | 1 | 2 |  |  |  |  |  |  |
| 2784 | 1 |  |  | 1 |  |  |  |  |
| 2814 | 1 |  |  | 1 |  |  |  |  |
| 2815 | 1 |  |  | 2 |  |  |  |  |
| 2816  Table S1 - continued. | 1 |  |  | 1 |  |  |  |  |
| 2817 | 1 |  |  | 1 |  |  |  |  |
| 2818 | 1 |  |  | 1 |  |  |  |  |
| 2819 | 1 |  |  | 1 |  |  |  |  |
| 2820 | 1 |  |  | 1 |  |  |  |  |
| 2821 | 1 |  |  | 1 |  |  |  |  |
| 3016 | 1 |  |  | 2 |  |  |  |  |
| 3109 | 3 |  |  |  | 1 |  |  |  |
| 3113 | 1 |  |  |  |  | 1 |  |  |
| 3122 | 2 |  |  |  | 1 |  |  |  |
| 3123 | 3 |  |  |  | 1 |  |  |  |
| 3124 | 3 |  |  |  | 1 |  |  |  |
| 3125 | 1 |  |  |  |  | 1 |  |  |
| 3129 | 1 |  |  | 1 |  |  |  |  |
| 3131 | 1 |  |  | 1 |  |  |  |  |
| 3132 | 1 |  |  | 1 |  |  |  |  |
| 3136 | 1 |  |  | 1 |  |  |  |  |
| 3141 | 1 |  |  | 1 |  |  |  |  |
| 3144 | 1 |  |  | 1 |  |  |  |  |
| 3147 | 1 |  |  | 1 |  |  |  |  |
| 3152 | 1 |  |  | 1 |  |  |  |  |
| 3160 | 1 |  |  | 1 |  |  |  |  |
| 3169 | 1 |  |  | 1 |  |  |  |  |
| 3176 | 1 |  |  |  |  | 1 |  |  |
| 3177 | 1 | 1 |  |  |  |  |  |  |
| 3179 | 3 |  |  |  | 1 |  |  |  |
| 3180 | 2 |  |  |  | 1 |  |  |  |
| 3181 | 3 |  |  |  | 1 |  |  |  |
| 3182 | 2 |  |  |  | 1 |  |  |  |
| 3304 | 2 |  |  |  | 4 |  |  |  |
| 3305 | 3 |  |  |  | 1 |  |  |  |
| 3309 | 3 |  |  |  | 5 |  |  |  |
| 3310 | 3 |  |  |  | 1 |  |  |  |
| 3312 | 2 |  |  |  | 1 |  |  |  |
| 3313 | 2 |  |  |  | 1 |  |  |  |
| 3314 | 2 |  |  |  | 1 |  |  |  |
| 3315 | 2 |  |  |  | 1 |  |  |  |
| 3316 | 2 |  |  |  | 1 |  |  |  |
| 3317 | 2 |  |  |  | 1 |  |  |  |
| 3318 | 2 |  |  |  | 1 |  |  |  |
| 3319 | 2 |  |  |  | 1 |  |  |  |
| 3323 | 2 |  |  |  | 1 |  |  |  |
| 3828 | 2 |  |  |  | 1 |  |  |  |

^1^ Clade was defined by the ST position on the CLONALFRAME genealogy (Figure 1).

^2^ Environmental isolates were cultured from duck (29), swan (2), pigeon (1) and gull (2) faeces and environmental water samples (33).
