# Supplementary material for: Evolution of an Agriculture-Associated Disease Causing Campylobacter coli Clade: Evidence from National Surveillance Data in Scotland
Source: PLoS One. 2010 Dec 15;5(12):e15708. doi: 10.1371/journal.pone.0015708 (PMC3002284; doi:10.1371/journal.pone.0015708)
Supplement: Table S2 — Allelic diversity at MLST loci of isolates from clinical and non-clinical datasets. (DOC) [file pone.0015708.s002.doc]

Table S2. Allelic diversity at MLST loci of isolates from clinical and non-clinical datasets.

|  | No. of alleles | | Alleles from clinical isolates present in non-clinical dataset (%) |
| --- | --- | --- | --- |
|  | Clinical isolates (500) | non-clinical isolates (1223) |  |
| *asp*A | 13 | 45 | 94 |
| *gln*A | 10 | 47 | 91 |
| *glt*A | 10 | 56 | 96 |
| *gly*A | 14 | 60 | 95 |
| *pgm* | 15 | 65 | 90 |
| *tkt* | 17 | 54 | 94 |
| *unc*A | 12 | 56 | 97 |
| ST | 103 | 393 | 85 |
